# Enhancing the Nutritional and Sensory Quality of Tartary Buckwheat Cookies Through Solid-State Fermentation with *Eurotium cristatum* and Baking

**DOI:** 10.3390/foods15040653

**Published:** 2026-02-11

**Authors:** Longyu Wan, Shuqi Liu, Xiao Wang, Zhibin Lv, Jianglin Zhao, Xiaoqin Zheng, Changying Liu, Wenjun Sun, Dabing Xiang, Liang Zou, Liangzhen Jiang

**Affiliations:** 1 School of Food and Biological Engineering, Chengdu University, Chengdu 610106, China; wanlongyu@cdu.edu.cn (L.W.); liushuqi@stu.cdu.edu.cn (S.L.); wangxiaowx@stu.cdu.edu.cn (X.W.); jlzhao@cdu.edu.cn (J.Z.); zhengxiaoqin@cdu.edu.cn (X.Z.); liuchangying@cdu.edu.cn (C.L.); xiangdabing@cdu.edu.cn (D.X.); 2 Sericulture Research Institute, Sichuan Academy of Agricultural Sciences, Nanchong 637099, China; 3 Department of Medical Instruments and Information, College of Biomedical Engineering, Sichuan University, Chengdu 610065, China; lvzhibin@pku.edu.cn; 4 Key Laboratory of Coarse Cereal Processing, Ministry of Agriculture and Rural Affairs, Sichuan Engineering and Technology Research Centre of Coarse Cereal Industrialization, Chengdu University, Chengdu 610106, China; sunwenjun@cdu.edu.cn (W.S.); zouliang@cdu.edu.cn (L.Z.)

**Keywords:** *Eurotium cristatum*, cookies, volatile components, fermentation, baking

## Abstract

Tartary buckwheat (*Fagopyrum tataricum*), a medicinal and edible crop, is valued for its richness in flavonoids and polyphenols, which confer antioxidant and hypoglycemic activities. *Eurotium cristatum*, a dominant fungus crucial for the quality of Fuzhuan tea, produces unique aromas and metabolites. This study developed cookies by replacing 20% of low-gluten flour with Tartary buckwheat flour that had undergone solid-state fermentation with *E. cristatum* followed by baking. Compared to cookies containing non-inoculated buckwheat flour, the fermented cookies contained significantly higher levels of total flavonoids (4.97 mg/g) and polyphenols (2.31 mg/g), and exhibited markedly enhanced antioxidant activity, as evidenced by a 16.4% higher ABTS radical scavenging rate and a 42.5% greater ferric reducing power. The fermented cookies also exhibited improved textural and sensory properties, a unique aroma profile characterized by pleasant floral notes, and a more homogeneous microstructure. HS-SPME-GC-MS analysis indicated that the optimized flavor resulted from the upregulation of key pleasant aroma compounds (e.g., (E)-2-nonenal, anethole) and the suppression of specific off-odor compounds (e.g., 2-ethyl-3,5-dimethylpyrazine, p-cresol). In conclusion, solid-state fermentation with *E. cristatum* followed by baking, effectively improves both the nutritional and sensory characteristics of Tartary buckwheat cookies, providing a viable strategy for developing novel, health-promoting bakery products with an appealing compelling flavor profile.

## 1. Introduction

*Eurotium cristatum*, also known as “Golden Flower” fungus, is a beneficial microorganism associated with dark tea. During the production of Fu brick tea, *E. cristatum* proliferates significantly to form the “Golden Flower” and produces a distinctive aroma, which is crucial for the tea’s unique flavor profile. Additionally, during fermentation, *E. cristatum* generates various bioactive metabolites such as alkaloids, piperazine derivatives, and fungal polysaccharides, which offer benefits including lipid reduction, gut microbiota modulation, antioxidant properties, and blood sugar regulation, thereby effectively modulating human metabolism [1]. Consequently, *E. cristatum* is also utilized in the biotransformation of various plant substrates to enhance sensory qualities and nutritional value.

Tartary buckwheat (*Fagopyrum tataricum*), an ancient cereal crop belonging to the Polygonaceae family, boasts both medicinal and edible properties. It encompasses seven major nutrients, including essential proteins, starch, and fiber, and is particularly rich in bioflavonoids such as rutin and quercetin [2], as well as polyphenols including catechins. Tartary buckwheat is often referred to as the “King of Grains” owing to its remarkable nutritional value. With the growing demand for “natural, nutritious, and healthy” foods, there is an increased interest in functional foods made from Tartary buckwheat. However, its bitter taste, coarse texture, and poor palatability limit its biological efficacy and the development of functional foods. Therefore, improving the processing quality of Tartary buckwheat is an urgent issue that needs to be addressed [3].

Fermentation can improve food flavor and enhance the nutrient content. *Monascus purpureus* and *E. cristatum* fermentation promotes the efficient release and enrichment of total phenols and flavonoids in Tartary buckwheat [4]. *Lactobacillus plantarum* fermentation of wheat increases the breakdown of crude protein, releasing various functional amino acids [5]. *E. cristatum* fermentation of oats [6], soybeans [7], ginkgo seeds [8] and common buckwheat, significantly increased the total phenol and flavonoid content, and enhanced their in vitro antioxidant activity. Fermented oats and common buckwheat also displayed increased inhibitory activities related to glucose and lipid metabolism [6,9], while fermented ginkgo seed powder suspension exhibits a pleasant aroma, with significantly improved amylase and protease activities [7]. *E. cristatum* fermented tartary buckwheat leaf tea presents a black tea flavor with a unique “fungus flower” aroma, improving sensory acceptance compared to unfermented tea [10]. Fermentation increases the content of volatile heterocyclic and aromatic compounds, enhances sweet flavor, and reduces acidic and bitter taste components. Therefore, *E. cristatum* is a promising candidate for Tartary buckwheat processing.

Despite established benefits of *Eurotium cristatum* fermentation in enhancing bioactive compounds and aroma in various substrates, its combined effect with baking in complex food matrices remains unexplored. This study aimed to address this gap by investigating the integration of *E. cristatum*-fermented and baked Tartary buckwheat flour into cookie production. We hypothesized that a 20% substitution with the fermented (whether or not subsequently baked) flour would optimize sensory acceptance while significantly increasing extractable phenolics and beneficially shifting the aroma profile toward more desirable floral and fruity notes. The primary objectives of this study were: to determine the optimal level of *E. cristatum*-fermented (whether or not subsequently baked) Tartary buckwheat flour incorporation in cookie formulation based on sensory evaluation; to comprehensively assess the impact of fermentation on the nutritional composition (e.g., total flavonoids, polyphenols) and in vitro antioxidant activity of the cookies; to characterize the volatile aroma profile of the fermented buckwheat flour and the optimized fermented cookies using HS-SPME-GC-MS and identify key aroma-active compounds contributing to the unique flavor; and to evaluate the effects of fermentation and baking on the textural properties and microstructure of the cookies.

## 2. Materials and Methods

### 2.1. Materials

Grains of the Tartary buckwheat variety ‘Chengku No. 2’ were provided by the Key Laboratory of Grain Processing, Ministry of Agriculture and Rural Affairs. The *E. cristatum* strain was previously isolated from Jinhua Zang fermented tea (Ya’an Zang Tea Co., Ltd., Ya’an, China), and preserved at CCTCC with the NO. M 20231875. Low-gluten flour, butter, salt, milk, and sugar were purchased from local markets. Commercial buckwheat cookies were acquired from Suqi Food Co., Ltd., Hai’an, China.

Reagents: Rutin standard, Folin-Ciocalteu reagent, gallic acid, DPPH, and ABTS(Yuanye Bio-Tech Co., Ltd., Shanghai, China). Aluminum chloride, potassium acetate solution, methanol, ether, ferric sulfate, hydrogen peroxide, salicylic acid, phosphate buffer, potassium ferricyanide, trichloroacetic acid, and ferric chloride (all analytical grade) (China National Pharmaceutical Group Chemical Reagent Co., Ltd., Shanghai, China).

### 2.2. Experimental Methods

#### 2.2.1. Preparation of *E. cristatum* Suspension

The preserved *E. cristatum* strain was first activated on potato dextrose agar (PDA) plates at 28 °C for 5–7 d. Subsequently, the culture was transferred to fresh PDA plates and incubated in the dark at 28 °C for 7–10 d to promote abundant sporulation, indicated by dense yellow mycelium with a powdery spore layer. Spores and attached mycelium were scraped into a 50 mL flask containing sterile water and glass beads (3–5 mm diameter), then shaken at 180 rpm for 30 min to release spores. The suspension was filtered through four layers of sterile gauze to remove hyphal debris. The spore concentration was determined using a hemocytometer and adjusted with sterile water to 1 × 10^7^–1 × 10^8^ spores/mL for subsequent fermentation experiments.

#### 2.2.2. Preparation Procedure of Buckwheat Flour

Tartary buckwheat grains were washed and dried in a hot-air dryer at 40 °C for 48 h [11]. The dried grains were ground and sieved through a 60-mesh screen to obtain untreated buckwheat flour.

Liquid-State Fermentation (LSF): UBF (25 g) was mixed with distilled water at a 1:3 ratio (*M*/*V*) in 250 mL Erlenmeyer flasks (100 mL working volume). The slurry was sterilized (121 °C, 20 min), and inoculated with a 5% (*V*/*M*) *E. cristatum* spore suspension. Fermentation proceeded in a reciprocating shaker at 180 rpm and 28 °C for 7 days. The fermented material was then dried at 40 °C and ground to obtain liquid-state fermented buckwheat flour.

Solid-State Fermentation (SSF): Whole buckwheat seeds (30 g) were mixed with distilled water at a 1:1 ratio (*M*/*V*) in 250 mL Erlenmeyer flasks, and sterilized (121 °C, 20 min). Following inoculation with 5% (*V*/*M*) *E. cristatum* spore suspension, the mixture was homogenized using a sterile spatula. Flasks were sealed with sterile breathable cotton plugs and incubated at 28 °C and 85% relative humidity for 7 days. The substrate was gently mixed once daily with a sterile spatula. All post-sterilization operations were conducted in a biosafety cabinet under aseptic conditions to maintain sterility. The absence of contamination was verified on days 3 and 7 by streaking 0.1 g of substrate onto PDA plates, incubating at 28 °C for 48 h, and visually inspecting for non-target microbial growth. The resulting solid-state fermented seeds (SSF-S) were collected for further processing.

Post-Fermentation Processing: To obtain different treated flours, the following processing steps were applied. For non-inoculated controls, sterilized seeds incubated under identical SSF conditions without inoculation were processed in two ways: dried at 40 °C for 2 h, then ground and sieved to produce untreated, non-inoculated buckwheat flour (NBF-U); high-temperature baked (170 °C, 15 min, then 90 °C, 30 min), then ground and sieved to produce non-inoculated, high-temperature baked buckwheat flour (NBF-B). For fermented materials, the SSF-S seeds were processed in two ways: dried at 40 °C for 2 h, then ground and sieved to produce untreated, fermented buckwheat flour (FBF-U); high-temperature baked (170 °C, 15 min, then 90 °C, 30 min), then ground and sieved to produce fermented and high-temperature baked Tartary buckwheat flour (FBF-B). All final flour samples were passed through a 60-mesh sieve before analysis.

#### 2.2.3. Preparation of Buckwheat Cookies

Buckwheat cookies were prepared using a standard formulation consisting of 29.5 g butter, 12 g milk, 8 g sugar, 0.5 g salt, and 50 g of a mixed flour blend (low-gluten flour and buckwheat flour). Butter was softened at 22 ± 2 °C, creamed (120 rpm, 3 min), then blended with sugar and salt (2 min). The flour-milk mixture was incorporated at low speed (60 rpm, 1 min) to form a homogeneous dough (23 ± 1 °C), which was rested at 4 °C for 15 min. The dough was piped (5 g per cookie) and baked in a convection oven at 160 ± 5 °C for 18 min [10], followed by cooling at 22 ± 2 °C for 30 min. To evaluate substitution levels, FBF-B was added at 0–40% (*w*/*w*) of the total flour mix. Based on preliminary tests, 20% substitution was selected for detailed comparison: fermented buckwheat cookies (FBC) contained 20% high-temperature baked fermented flour, while non-inoculated buckwheat cookies (NBC) contained 20% untreated, non-inoculated buckwheat flour. Commercial buckwheat cookies (CM) were used as a market reference.

#### 2.2.4. Sensory Evaluation

Sensory evaluation was conducted by ten trained panelists (5 females, 5 males; aged 18–45 years) with no history of taste or olfactory disorders and prior experience in cereal-based bakery assessment. Panelists completed a 3-day training program (2 h/day) to familiarize themselves with the sensory attributes defined in Table 1 (morphology, flavor, crispness, color, and organizational structure) and to calibrate scoring using reference samples (commercial, unfermented, and fermented buckwheat cookies). Evaluations were performed in individual booths under white fluorescent lighting at controlled temperature (22 ± 2 °C) and humidity (50 ± 5%). Each sample (5 g) was coded with a random 3-digit number and presented in a randomized order. Palate cleansers (deionized water and unsalted crackers) were provided between samples. All samples were assessed in triplicate across three independent production batches (*n* = 3). For each batch, the scores from the ten panelists were averaged to obtain a batch-level score; the final sensory result for each attribute is reported as the mean ± standard deviation of the three batch-level scores.

#### 2.2.5. Analysis of Basic Nutrients

The contents of moisture, crude fat, total starch, and crude protein in the buckwheat cookie samples were determined in triplicate according to the corresponding Chinese national food safety standards. Moisture was analyzed by the direct drying method (GB 5009.3-2016) [12], crude fat by Soxhlet extraction (GB 5009.6-2016) [13], total starch by acid hydrolysis (GB/T 5009.9-2023) [14], and crude protein by the Kjeldahl method (GB 5009.5-2016) [15] using a nitrogen-to-protein conversion factor of 6.25. Results are expressed as mean ± standard deviation.

#### 2.2.6. Determination of Total Flavonoid and Polyphenol Content

Total flavonoid content (TFC) was determined using an aluminum chloride colorimetric method [16]. Briefly, samples (0.2 g) were extracted with 10 mL of 70% methanol under ultrasonication, centrifuged, and the extraction repeated. The combined supernatant was reacted with AlCl_3_ (0.1 M) and CH_3_COOK (1 M), incubated in the dark for 30 min, and the absorbance measured at 420 nm. TFC was calculated from a rutin standard curve and expressed as rutin equivalents per gram. Total polyphenol content (TPC) was assessed using the Folin-Ciocalteu method [17]. Samples were similarly extracted, and the supernatant was diluted, mixed with Folin-Ciocalteu reagent and Na_2_CO_3_ solution, incubated at 40 °C for 1 h, and the absorbance measured at 760 nm. TPC was calculated from a gallic acid standard curve and expressed as gallic acid equivalents per gram.

#### 2.2.7. Color Measurement

Cookie color was measured using a colorimeter (NR10QC, Guandong Threenh Technology Co., Ltd., Shenzhen, China) calibrated with a white reference plate. Color values were recorded in the CIE Lab color space, with L* (lightness), a* (redness/greenness), and b* (yellowness/blueness) representing the surface color of the samples.

#### 2.2.8. Texture Analysis

Texture analysis was performed on cookies after 1 h of cooling at room temperature. Samples from the central region of each cookie (thickness: 5.0 ± 0.2 mm; diameter: 60 ± 2 mm) were placed horizontally on the test platform of a texture analyzer (TA.XT plus, Ruifeng Intelligent Technology Co., Ltd., Shanghai, China) equipped with a TA9 probe. The test settings were: pre-test speed 2 mm/s, test speed 1 mm/s, post-test speed 2 mm/s, trigger force 5 g, compression distance 2 mm. Hardness was defined as the maximum force (N) at 2 mm compression; chewiness (N) was calculated as the product of hardness, springiness, and cohesiveness. Each sample group was analyzed in triplicate, and mean values are reported.

#### 2.2.9. Flavor Analysis by Electronic Tongue

Taste profiles were analyzed using an Astree electronic tongue system (Alpha M.O.S., Toulouse, France). Ground cookie samples (5.0 g) were extracted with 150 mL of boiling distilled water for 10 min, vacuum-filtered while hot, and the filtrate was cooled to 22 ± 2 °C and adjusted to its original volume with distilled water. A 100 mL aliquot of the prepared solution was transferred to an autosampler vial for measurement. Each sample was analyzed with six technical replicates (120 s acquisition per measurement), and sensors were rinsed with deionized water for 60 s between samples to prevent carryover. Prior to analysis, the sensor array was activated in deionized water for 30–60 min, and the system was calibrated with 0.01 mol/L HCl. Measurements were performed on three independent biological batches (*n* = 3), and the average of the technical replicates for each batch was used for data processing.

#### 2.2.10. Microstructure Analysis

The microstructure of the cookie cross-sections was examined using a scanning electron microscope (Apreo 2C, Thermo Fisher Scientific, Waltham, MA, USA). Freeze-dried cookie samples were cryo-fractured in liquid nitrogen to expose fresh cross-sections, sputter-coated with gold, and observed at an accelerating voltage of 3.00 kV and a magnification of 5000×.

#### 2.2.11. In Vitro Antioxidant Capacity Measurement

For antioxidant assays, a sample stock solution was prepared by accurately weighing 0.5 g of cookie powder into 5 mL of 70% methanol. The mixture was ultrasonicated (40 kHz, 150 W, 50% duty cycle) at room temperature for 30 min, then centrifuged at 4000 rpm for 10 min. The extraction was repeated twice on the residue under the same conditions. The combined supernatants were adjusted to a final volume of 15 mL with 70% methanol, yielding a stock solution of 100 mg/mL.

##### DPPH Radical Scavenging Activity

The DPPH radical scavenging activity was measured as described previously with slight modifications [18]. Briefly, 2 mL of sample solution was mixed with 2 mL of 0.1 mmol/L DPPH solution (in absolute ethanol), vortexed, and incubated in the dark at room temperature for 30 min. The absorbance was measured at 517 nm (*A_i_*). Control samples containing 70% methanol instead of DPPH solution (*A_j_*) or instead of sample solution (*A_c_*) were included. A 20 µg/mL ascorbic acid solution served as the positive control. All assays were performed in triplicate, and the scavenging activity (%) was calculated as:

(1) $$\mathrm{DPPH}\;\mathrm{radical}\;\mathrm{scavenging}\;\mathrm{rate}\;(\%)=(1-\frac{A_{i}-A_{j}}{A_{c}})\times 100\%$$

##### ABTS Radical Scavenging Activity

ABTS radical scavenging activity was assessed as described previously with slight modifications [19]. The ABTS working solution was prepared by mixing equal volumes of 7 mmol/L ABTS solution and 2.5 mmol/L potassium persulfate, followed by incubation in the dark at room temperature for 24 h. The resulting stock solution was diluted with 95% ethanol to an absorbance of 0.70 ± 0.02 at 734 nm. For the assay, 0.1 mL of the diluted sample was mixed with 3.9 mL of the ABTS working solution, incubated in the dark for 6 min, and the absorbance was measured at 734 nm (*A_i_*). Appropriate controls included substituting the ABTS solution with ethanol (*A_j_*) and the sample with 70% methanol (*A_c_*). A 1 mg/mL ascorbic acid solution served as a positive control. All determinations were performed in triplicate. The scavenging activity (%) was calculated as:

(2) $$\mathrm{ABTS}\;\mathrm{radical}\;\mathrm{scavenging}\;\mathrm{rate}\;(\%)=(1-\frac{A_{i}-A_{j}}{A_{c}})\times 100\%$$

##### OH Radical Scavenging Activity

Hydroxyl radical scavenging activity was determined according to a reported method with slight modifications [20]. Briefly, 0.5 mL of sample solution was mixed sequentially with 0.5 mL of 9 mmol/L FeSO_4_ and 0.5 mL of 7% H_2_O_2_. After incubation in the dark at room temperature for 10 min, 0.5 mL of 6 mmol/L salicylic acid (in ethanol) was added, and the mixture was further incubated at 37 °C in the dark for 30 min. The absorbance was measured at 510 nm (*A_i_*). Controls were prepared by replacing H_2_O_2_ with distilled water (*A_j_*) or the sample with 70% methanol (*A_c_*). A 2 mg/mL ascorbic acid solution was used as the positive control. The assay was performed in triplicate, and the scavenging activity (%) was calculated as:

(3) $$\mathrm{OH}\;\mathrm{radical}\;\mathrm{scavenging}\;\mathrm{rate}\;(\%)=(1-\frac{A_{i}-A_{j}}{A_{c}})\times 100\%$$

##### Ferric Reducing Antioxidant Power (FRAP)

The reducing power was determined by the ferricyanide reduction method [19]. Briefly, 0.2 mL of sample solution was mixed with 1 mL of 0.2 mol/L phosphate buffer (pH 6.6) and 1 mL of 1% potassium ferricyanide. After incubation at 50 °C in the dark for 20 min, 1 mL of 10% trichloroacetic acid was added, and the mixture was centrifuged at 4000 rpm for 10 min. Subsequently, 1 mL of the supernatant was combined with 0.2 mL of 1 mg/mL FeCl_3_ solution and allowed to stand in the dark for 10 min. The absorbance was measured at 700 nm (*A*_1_), with 70% methanol serving as the blank (*A*_2_). A 1 mg/mL ascorbic acid solution was used as a positive control. The assay was performed in triplicate. The reducing power is calculated using the following formula:

(4) $$\mathrm{Reducing}\;\mathrm{power}=A_{1}-A_{2}$$

#### 2.2.12. Analysis of Volatile Aroma Compounds

Volatile compounds were extracted from cookie samples by automated headspace solid-phase microextraction (HS-SPME) coupled with gas chromatography–mass spectrometry (GC–MS) [21]. Approximately 500 mg of finely ground sample, 2 mL of saturated NaCl solution, and 20 µL of internal standard solution (2-octanol, 10 µg/mL) were sealed in a 20 mL headspace vial. Prior to extraction, a 120 µm DVB/CAR/PDMS SPME fiber was conditioned at 250 °C for 5 min. The sample was equilibrated at 60 °C for 5 min with agitation, followed by headspace extraction for 15 min. The fiber was then thermally desorbed in the GC injector at 250 °C for 5 min. Separation was performed on a DB-5MS capillary column (30 m × 0.25 mm × 0.25 µm, Agilent J&W Scientific, Folsom, CA, USA) with high-purity helium (99.999%) as the carrier gas at a constant flow of 1.2 mL/min. The oven temperature was programmed as follows: 40 °C (hold 3.5 min), increased to 100 °C at 10 °C/min, then to 180 °C at 7 °C/min, and finally to 280 °C at 25 °C/min (hold 5 min). The injector was operated in spitless mode at 250 °C.

Mass Spectrometry Conditions: The mass spectrometer was operated in electron ionization (EI) mode at 70 eV. The ion source, quadrupole, and transfer line temperatures were maintained at 230 °C, 150 °C, and 280 °C, respectively. Data were acquired in selected ion monitoring (SIM) mode, targeting specific qualitative and quantitative ions for accurate identification and semi-quantification. The relative content of each compound (*X_i_*, μg/g) was calculated using the internal standard method according to the following formula:

(5) $$X_{i}=\frac{V_{s}\times C_{s}}{M}\times \frac{I_{i}}{I_{s}}\times 10^{-3}$$

where: *X_i_* represents the concentration of compound *i* in the sample (μg/g); *V_s_* is the volume of the internal standard added (μL); *C_s_* is the concentration of the internal standard (μg/mL); *M* is the amount of the sample (g); *I_s_* is the peak area of the internal standard; and *I_i_* is the peak area of compound *i* in the sample.

#### 2.2.13. Relative Odor Activity Value (rOAV) Calculation

The *rOAV* was used to identify key aroma-active compounds in the samples by relating their concentrations to their sensory impact [22,23]. The *rOAV* for each compound was calculated as follows:

(6) $${rOAV}_{i}=\frac{V_{i}}{T_{i}}$$

where *rOAV_i_* is the relative odor activity value of compound *i*, *C_i_* is its concentration in the sample, and *T_i_* is its odor threshold in water.

Odor thresholds (*T_i_*) were primarily sourced from authoritative compilations [24], databases (e.g., www.vcf-online.nl, accessed on 15 October 2025), and recent literature on food aroma [25,26,27]. For compounds with multiple reported values, the most frequently cited and recent threshold was selected to ensure consistency. A compound was considered a significant aroma contributor if its *rOAV* was ≥1.

### 2.3. Data Analysis

Data are presented as the mean ± standard deviation of at least three independent replicates. Statistical analysis was performed using SPSS Statistics 26.0 (IBM, Armonk, NY, USA), with comparisons among groups conducted by one-way analysis of variance (ANOVA) followed by Duncan’s multiple range test. Differences were considered statistically significant at *p* < 0.05. Data organization and preliminary calculations were carried out in Excel 2020 (Microsoft, Redmond, WA, USA), and graphs were generated using Origin 2022b (OriginLab, Northampton, MA, USA).

## 3. Results and Discussion

### 3.1. Selection of Processing Methods for Buckwheat Flour

**Figure 1:**
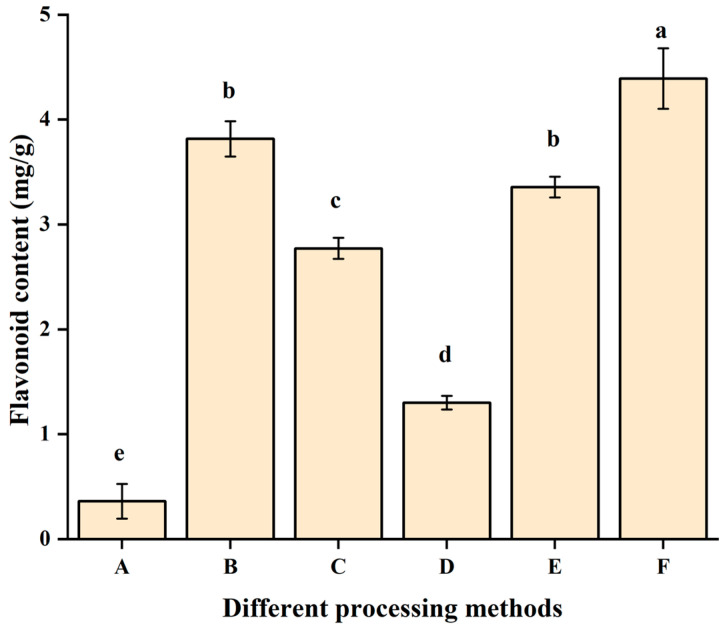
Effects of different processing methods of Tartary buckwheat flour on the total flavonoid content of cookies. A: control (100% low-gluten flour). B: untreated buckwheat flour. C: untreated, non-inoculated buckwheat flour. D: liquid-state fermented buckwheat flour. E: untreated, fermented buckwheat flour. F: high-temperature baked fermented buckwheat flour. Values with different lowercase letters indicate significant differences between groups (*p* < 0.05).

To evaluate the influence of incorporating fermented and/or baked Tartary buckwheat powder on traditional cookie quality, the high-yielding cultivar ‘Chengku No. 2’ (*Fagopyrum tataricum*) was selected as the raw material for its strong promotion potential in Sichuan Province and suitability for food processing [28]. The *E. cristatum* strain CCTCC M 20231875, previously isolated from Jinhua Zang fermented tea, was used as the starter culture, owing to its documented ability to enhance flavonoid and polyphenol contents and degrade starch and lipids [29]. Given that buckwheat flour processing significantly affects the cookie quality [30], four pretreatment methods were applied to the flour, each incorporated at 20% into the cookie formulation. Pretreatment markedly influenced the total flavonoid content of the cookies, as shown in Figure 1. The control cookie with 100% low-gluten flour contained 0.36 mg/g of flavonoid only, while the addition of 20% of untreated buckwheat flour increased the flavonoid content significantly to 3.81 mg/g (Figure 1B). Autoclaving reduced the flavonoid’s content to 2.77 mg/g (Figure 1C), consistent with thermal degradation of heat-sensitive compounds such as rutin and quercetin [31]. Liquid fermentation by *E. cristatum* further decreased the content to 1.29 mg/g (Figure 1D). In contrast, solid-state fermentation by *E. cristatum* restored the flavonoid content to 3.35 mg/g, while high-temperature baking after solid-state fermentation yielded the highest content (Figure 1F, 4.39 mg/g). The flavonoid reduction in liquid fermentation may result from extensive oxygenation and rapid fungal growth leading to nutrient depletion [32,33,34,35]. Conversely, solid-state fermentation enables enzymatic hydrolysis of phenolic-cell wall bonds, releasing insoluble compounds and enhancing bioavailability [36,37]. The observed increase in group F may be partially attributed to Maillard reaction products interfering with the colorimetric assay, though carbon utilization assays were not conducted. The metabolic activity of *E. cristatum* has been shown to effectively reduce bitterness and astringency in various plant matrices, such as tree peony flower tea and mulberry leaf tea, by modulating metabolites like catechins and proanthocyanidins [38,39]. While the spectrophotometric data require validation using HPLC, sensory evaluation confirmed that baking after solid-state fermentation effectively reduced astringency and improved acceptability (Section 3.2). The high-temperature baking step, while crucial for product development, may also induce thermal degradation of certain compounds. Thus, solid-state fermentation with *E. cristatum* followed by baking (160–180 °C) is recommended as a promising pretreatment for buckwheat flour in cookie production, effectively enhancing both nutritional and sensory properties.

### 3.2. Sensory Evaluation of Cookies

**Figure 2:**
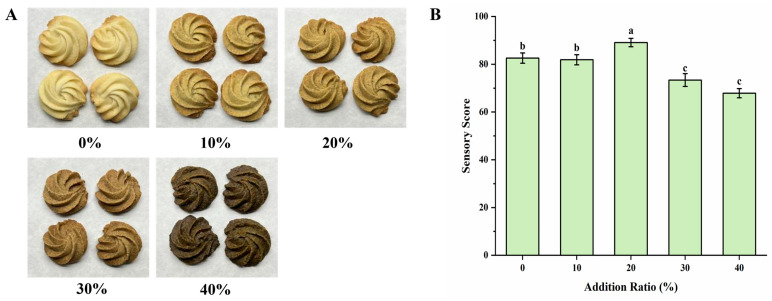
Effects of substitution levels of fermented Tartary buckwheat flour on the appearance and sensory evaluation of cookies. (**A**) Physical appearance of cookies prepared with different substitution levels of fermented buckwheat flour. (**B**) Sensory evaluation scores of cookies with varying substitution levels. Data are presented as mean ± SD (*n* = 3). Different lowercase letters indicate significant differences (*p* < 0.05).

Sensory evaluation serves as a key indicator for assessing cookie quality [40]. To examine the effect of fermented Tartary buckwheat flour on the sensory attributes of cookies, a sensory evaluation form was developed (Table 1) and products were scored based on flavor and other characteristics. As shown in Figure 2, the addition level of fermented flour significantly influenced cookie appearance and sensory scores. Since buckwheat flour is naturally white with a greenish tinge and fermentation with *E. cristatum* further deepens the color, a progressive darkening of the cookies was observed with increasing substitution levels (Figure 2A). At 10% addition, cookies were darker than the control (0% addition), yet without significant sensory differences; the fermented flavor was mild and bitterness was subtle. With 20% fermented flour, cookies were darker than the 10% group, exhibiting a balanced bitterness, a distinct fermented aroma, and a rich texture. This group received the highest sensory score of 89.1, significantly outperforming other formulations (Figure 2B). At 30% addition, cookies turned darker with a strong fermented aroma, pronounced bitterness, and slight sourness, leading to inferior texture and flavor, and a lower score than the control. At 40%, the color further darkened, resembling dark chocolate, with intensified bitterness and worsened flavor despite a strong aroma. In summary, cookies with 20% fermented buckwheat flour achieved the best sensory acceptance.

### 3.3. Color Analysis of Cookies

Cookie color is an important indicator of product quality [40]. The effect of adding different amounts of fermented buckwheat flour on the color parameters of cookies is summarized in Table 2. As the proportion of fermented flour increased, the lightness (L* value) of the cookies decreased progressively from 70.18 ± 0.213 (control) to 49.37 ± 0.074 (40% substitution), indicating a gradual darkening in appearance. No clear trend was observed for the redness-greenness (a* value). In contrast, the blueness-yellowness (b* value) also declined steadily from 22.32 ± 0.236 to 15.21 ± 0.153 with increasing substitution level, further supporting the observed darkening effect. These color measurements are consistent with the visual appearance of the cookies shown in Figure 2A. The darkening of the cookies is likely a result of multiple non-enzymatic and microbial metabolic reactions [41,42,43]. Specifically, Maillard reactions (between reducing sugars and amino acids) and caramelization (thermal decomposition of sugars) are well-known to contribute to the browning of baked products [44]. In addition, microbial metabolic processes during *E. cristatum* fermentation always generate colored metabolites [45], though further targeted analyses would be required to confirm the presence and identity of such compounds.

### 3.4. Texture Analysis of Cookies

Cookie texture is a key parameter for evaluating cookie quality [46]. The textural properties of cookies prepared with different substitution levels of fermented buckwheat flour are summarized in Table 3. At 10% substitution, cookie hardness (356.95 ± 4.93) was lower than that of the control (469.62 ± 28.47), while chewiness was significantly improved. Hardness increased with higher substitution levels, reaching a maximum at 30% addition (655.67 ± 13.16), approximately 1.40 times that of the control. However, at 40% substitution, hardness decreased to 523.15 ± 18.23, though it remained higher than the control. Chewiness was significantly enhanced at all substitution levels above 10%, with values exceeding 1.75 times that of the control. These results indicate that the incorporation of fermented and baked buckwheat flour improves chewiness and modulates hardness in a dose-dependent manner. A 20% substitution level provided a balanced improvement in both hardness and chewiness. The enhanced chewiness, a textural parameter associated with a more satisfying and cohesive mouthfeel [47], along with an optimally modulated hardness that provides a desirable crispness rather than excessive brittleness, directly contributed to the higher sensory acceptance scores [48].

### 3.5. Flavor Evaluation of Cookies

**Figure 3:**
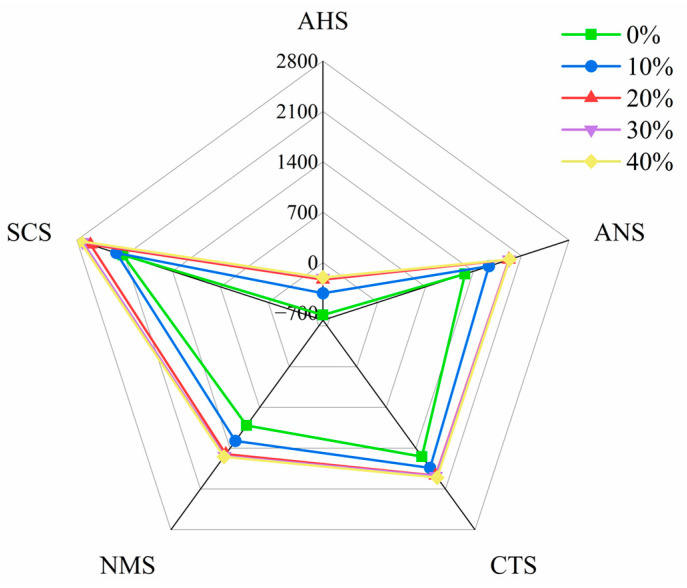
Taste profile of cookies with different amounts of buckwheat flour added (AHS, sourness; ANS, sweetness; CTS, saltiness; NMS, umami; and SCS, bitterness).

Flavor is a critical attribute determining cookie quality [49]. The taste profiles of cookies containing different substitution levels of fermented buckwheat flour were analyzed using an electronic tongue (Figure 3). The sensor attributes are denoted as follows: AHS, sourness; ANS, sweetness; CTS, saltiness; NMS, umami; and SCS, bitterness. The results show that as the proportion of fermented buckwheat flour increased up to 20%, the saltiness, umami, sweetness, and bitterness of the cookies increased significantly, while sourness decreased. Beyond 20%, no notable changes were observed in umami, sweetness, or saltiness. Owing to the high content of bitter compounds such as flavonoids in fermented buckwheat flour, bitterness increased with higher substitution levels. Cookies with 20% fermented flour exhibited lower bitterness than those with 30% or 40% substitution, though slightly higher than the 10% group. Moreover, the 20% formulation displayed balanced umami, saltiness, and sweetness, along with a distinct pleasant fermented aroma characterized by strong and pleasant fungi-flowery notes, which contributed to higher sensory acceptance. Thus, a 20% addition of fermented buckwheat flour is recommended for optimal flavor quality in fermented buckwheat cookies.

### 3.6. Moisture and Nutritional Component Analysis of Cookies

To further evaluate the impact of *E. cristatum* fermentation on the nutritional quality of buckwheat cookies, a comparative analysis was conducted among three types of cookies: commercial buckwheat cookies (CM), non-inoculated buckwheat cookies (NBC), and fermented buckwheat cookies (FBC). The basic nutritional composition (fat, starch, crude protein) and bioactive compounds (total flavonoids and total polyphenols) were determined, and the results are summarized in Table 4. Compared with NBC, the moisture and fat content of FBC showed no significant changes, complying with the requirements of the Chinese national standard GB/T 20980-2021 [50]. Both buckwheat cookies (fermented and non-inoculated) exhibited higher fat content than commercial cookies, which can be attributed to the higher butter content used in the formulation. After fermentation and baking, the starch and total protein content decreased significantly, falling below the levels in CM, especially the fermented group (34.10 ± 0.30 vs. 46.60 ± 0.89 for starch, and 5.31 ± 0.01 vs. 7.92 ± 0.02 for crude protein). The addition of buckwheat flour significantly increased the total flavonoid (4.00 ± 0.05 vs. 1.86 ± 0.09 mg/g) and polyphenol (1.67 ± 0.07 vs. 0.74 ± 0.03 mg/g) contents compared to commercial cookies. Notably, fermentation and baking further increased the content of these bioactive compounds. Fermented buckwheat cookies contained 4.97 ± 0.13 mg/g of flavonoids and 2.31 ± 0.06 mg/g of total polyphenols, which are about 24.25% and 38.32% higher than the untreated, non-inoculated group respectively.

The enhanced nutritional profile of buckwheat cookies—marked by increased total flavonoid and polyphenol content and reduced starch and protein levels—is primarily attributable to solid-state fermentation with *E. cristatum*. This bio-enhancement aligns with the documented ability of the fungus to enrich bioactive compounds in substrates including ginkgo leaves [51] and seeds [52], soybeans [7,53], oats [6], and buckwheat [4,9]. and can be explained by its enzymatic activity. The observed biochemical changes may be facilitated by the enzymatic potential of *E. cristatum* [54]. Our prior work with this strain demonstrated a significant increase in the activity of key enzymes during fermentation, including phenylalanine ammonia-lyase (PAL) and polyphenol oxidase (PPO), alongside genomic confirmation of the corresponding genes [55]. The mechanism involves a dual action: hydrolytic enzymes (e.g., α-amylase, proteases) break down macromolecules, while enzymes like β-glucosidase potentially release bound phenolics [4,45]. Concurrently, biosynthetic enzymes such as PPO are implicated in the biotransformation and accumulation of bioactive compounds. This positions fermentation as an effective biocatalytic strategy for developing functional foods, though further targeted omics studies are needed for definitive pathway validation.

### 3.7. Microscopic Structure of Cookies

**Figure 4:**
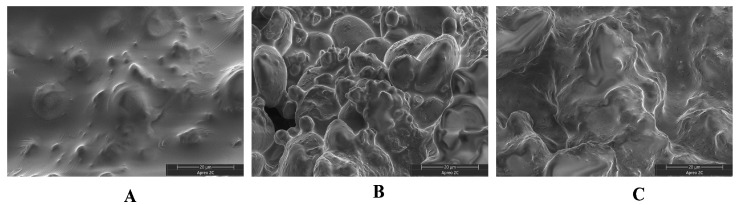
Electron microscope scan of biscuit section. (**A**) CM, Commercial Buckwheat Cookies; (**B**) NBC, Non-inoculated Buckwheat Cookies; (**C**) FBC, Fermented Buckwheat Cookies.

To further investigate the impact of *E. cristatum* fermentation on cookie texture, the microstructure of cross-sections of the three cookie types was examined using scanning electron microscopy (SEM). As shown in Figure 4, all cookies exhibited densely packed starch granules on the surface, which may be attributed to starch-lipid complex formation during baking [56]. CM showed the smoothest cross-section among the samples (Figure 4A), with fewer protrusions and minimal internal particles, along with a relatively uniform and firm gluten network. In contrast, NBC displayed more surface protrusions and internal particles, greater structural discontinuity, and although starch granules remained encapsulated, the matrix showed noticeable relaxation and the presence of small voids [57]. These pores likely trap air, during dough mixing and baking leading to surface protrusions and increased internal particles, thereby contributing to a poorer texture. FBC also exhibited surface protrusions and internal particles, but to a lesser extent than NBC, along with reduced structural damage and improved surface smoothness, which is consistent with the texture results presented in Table 4. The reduction in internal particles and improved structural integrity in fermented cookies may be mechanistically linked to compositional and processing-related shifts induced by *E. cristatum* fermentation. Specifically, fermentation-mediated enzymatic hydrolysis of starch (generating smaller starch degradation products) and partial protein degradation (Table 4), likely reduced the size of insoluble particulates in the dough [58]. Additionally, the potential alteration of the composition and functionality of dietary fiber derived from fermented buckwheat may help regulate water distribution and molecular interactions within the dough matrix [59]. This regulation likely promotes more uniform starch gelatinization during baking and reduces void formation. These changes—coupled with mild gluten network dilution by fermented flour components—avoided excessive gluten cross-linking, resulting in a more homogeneous matrix with fewer aggregated particles. This aligns with previous findings that fungal-fermented dietary fiber modulates gluten network formation to reduce structural heterogeneity [4]. Microstructural optimization further translated to improved sensory-relevant texture properties (Table 4). Unlike the non-inoculated group, which exhibited excessive crumbliness and uneven bite force, fermented cookies showed balanced shortness (friability) and surface smoothness—key quality attributes for cookie acceptability. This alignment between microstructure (uniform matrix, reduced voids/particles) and texture performance underscores that *E. cristatum* fermentation modulates cookie quality through targeted structural modifications, meeting consumer expectations for crisp, smooth-textured cookies.

### 3.8. Analysis of Volatile Profiles of Cookies

#### 3.8.1. Analysis of Total Volatile Components

**Figure 5:**
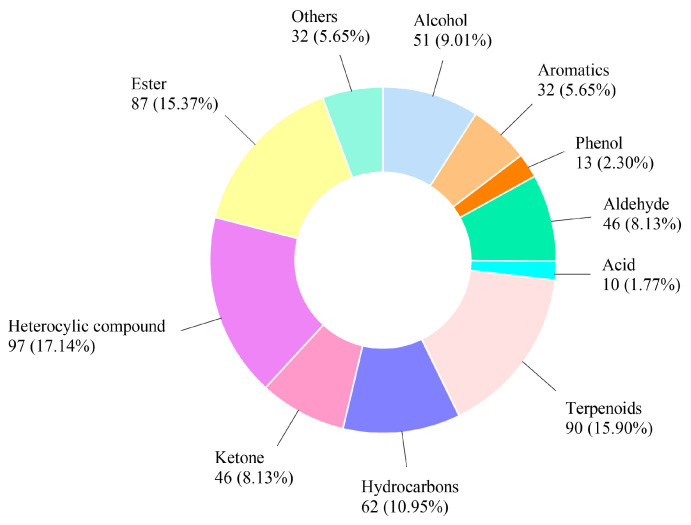
Pie chart of total volatile substances.

As mentioned earlier, cookies made with baked and fermented buckwheat flour by *E. cristatum* emit a strong and pleasant mushroom-flowery aroma, significantly differing from the non-inoculated and commercial buckwheat cookies. This suggests its potential as a novel, unique-flavored functional cookie. Headspace solid-phase microextraction-gas chromatography-mass spectrometry (HS-SPME-GC/MS) was employed to analyze the composition of the cookie aroma. To compare the effects of *E. cristatum* fermentation and/or baking (170 °C, 15 min, then 90 °C, 30 min) on the aroma components of buckwheat flour and cookies respectively, a total of 7 sample groups dividing into two major categories were analyzed. The first category included different treatments of buckwheat flour—untreated and non-inoculated buckwheat flour (NBF-U), non-inoculated, baked buckwheat flour (NBF-B), untreated, fermented buckwheat flour (FBF-U), and fermented, baked buckwheat flour (FBF-B). The second category comprised 3 types of cookies, CM, NBC, and FBC. As shown in Figure 5, totally 566 volatile organic compounds (VOC) across 11 major categories were identified. Heterocyclic compounds were the most prevalent, comprising approximately 17.1% of the total volatiles, followed by terpenoids (15.90%), ester (15.37%), hydrocarbons (10.95%), alcohol (9.01%), aldehyde (8.13%), ketone (8.13%), aromatics (5.65%), others (5.65%), phenol (2.30%), and acid (1.77%).

#### 3.8.2. Impact of Baking and Fermentation on Aroma-Active Compounds of Buckwheat Flour

The volatile profiles of four buckwheat flour groups were analyzed based on compound content and relative Odor Activity Value (rOAV) to evaluate the effects of baking and fermentation. A total of 49 volatile compounds with a fold change >1 in both content and rOAV values across comparisons were identified as key aroma-active compounds, and their rOAV values are summarized in Table 5. Baking significantly altered the volatile composition of both non-inoculated and fermented buckwheat flour. Compared to NBF-U, the baked counterpart (NBF-B) showed significant content changes (|log_2_FC| > 1) in 50 compounds, 8 of which had significant rOAV differences. Key up-regulated compounds included (+)-α-pinene, 1-octen-3-ol, 1-octen-3-one, 2-acetylthiazole, and (isothiocyanatomethyl) benzene, while anethole, 10-undecenal, and 2,2,4-trimethyl-1,3-pentanediol diisobutyrate were down-regulated. Notably, the rOAV of 1-octen-3-one (mushroom-like aroma) increased dramatically from 2360.90 to 11,080.32, identifying it as a key baking-generated aroma, likely from thermal degradation of lipids or amino acids [60].

In the fermented group, baking induced even more pronounced changes. Comparing FBF-B to FBF-U revealed 140 compounds with significant content changes, 31 of which showed |log_2_FC| > 1 in rOAV. Among 11 up-regulated compounds (Table 5), butanoic acid, 3-methylbutanoic acid 2-phenylethyl ester (rOAV = 237.44) and α-irone contributed fruity and floral notes, while dimethyl trisulfide (rOAV increased from 8774.47 to 22,114.80) imparted a savory, sulfury aroma. Conversely, 20 compounds were down-regulated, including aldehydes such as (2E,4Z)-2,4-decadienal, 2-nonenal, (E)-2-nonenal, and (E)-4-decenal (fatty, green/waxy notes) and pyrazines such as 2-ethyl-3,5-dimethylpyrazine (burnt, roasted aroma), suggesting a refinement of the overall aroma profile.

To further elucidate the specific effect of *E. cristatum* fermentation on flavor, the volatile profiles of buckwheat flour with and without fermentation were directly compared. Fermentation induced profound and consistent changes in the volatile composition, irrespective of subsequent baking. A significantly greater number of volatile compounds were altered by fermentation (184 in FBF-U vs. NBF-U; 270 in FBF-B vs. NBF-B) compared to those affected by baking alone, underscoring its dominant role in aroma transformation. In the comparison of unbaked flours (FBF-U vs. NBF-U), 21 up-regulated compounds exhibited an rOAV fold-change > 2. These included key pleasant aroma contributors such as the aldehydes 10-Undecenal and benzeneacetaldehyde (sweet, floral), the aromatics anethole and trans-anethole (sweet, licorice-like), the ester methyl benzoate (floral, fruity), and the ketone 1-octen-3-one (mushroom-like). Concurrently, compounds associated with undesirable odors—including the fatty/alcoholic 1-nonanol, the burnt/pyrazines 2-ethyl-3,5-dimethylpyrazine, and the animalic p-cresol—were significantly down-regulated.

A similar, albeit different, up-regulation pattern was observed in the baked flour comparison (FBF-B vs. NBF-B), with 22 up-regulated compounds showing an rOAV fold-change > 2. These include aldehydes (6), aromatics (2), esters (5), ketones (2), terpenoids (2), heterocyclic compounds (2), sulfur compounds (2) and alcohols (1), many of them exhibit pleasant sweet, flowery, or fruity aromas (Table 5). Among them, those with high rOAV values (>50) in FBF-B were aldehydes including esters including 2,2,4-trimethyl-1,3-pentanediol diisobutyrate and 2-propenoic acid, ethyl ester, aldehydes including 10-undecenal, (E,E)-2,6-nonadienal, (E,Z)-2,6-nonadienal, (Z)-6-nonenal, the aromatics anethole, the alcohol 1-octen-3-ol, the sulfur compounds (isothiocyanatomethyl)-benzene, dimethyl trisulfur compounds. Notably, the sulfur compound dimethyl trisulfide compounds, imparting a savory, onion-like meaty aroma, reached an exceptionally high rOAV (22,114.8) only in the fermented and baked flour (FBF-B), which may be one of the key contributors to the unique flavor of the fermented cookies. Meanwhile, an expanded set of unpleasant grassy, green, and burnt compounds (e.g., p-Cresol, dodecanenitrile, (E,E)-3,5-octadien-2-one, 2-ethyl-3,5-dimethylpyrazine) were down-regulated in FBF-B.

The volatile changes observed in this study demonstrate that fermentation with *E. cristatum* is the predominant driver of aroma transformation in Tartary buckwheat flour, while baking acts primarily as a modulator and intensifier of the fermented volatile profile. Fermentation fundamentally reshaped the aroma composition by orchestrating a beneficial shift: it consistently up-regulated key compounds contributing desirable sweet, floral, and fruity notes—such as anethole, methyl benzoate, and 10-undecenal—while simultaneously down-regulating compounds responsible for green, grassy, and burnt off-notes, including specific aldehydes and pyrazines. The ability of *E. cristatum* to beneficially modulate aroma profiles—enhancing pleasant notes while suppressing off-flavors—has been demonstrated across various substrates, including co-fermented black Tartary buckwheat [4], bamboo leaves [61], and Fu brick tea [62], highlighting its consistent flavor-shaping role through microbial metabolism.

The interaction between fermentation and baking is complex. Previous studies indicate that baking can enhance the flavor and nutrition of fermented products [63,64,65,66], the interaction observed here is more complex, not merely additive. This interaction manifested in two distinct patterns. First, a non-additive effect was observed for certain compounds: although fermentation upregulated the fungal metabolites 1-octen-3-one and 1-octen-3-ol, baking the fermented flour did not further increase their levels—and in some cases even reduced them—despite their significant thermal generation in non-inoculated flour. Second, a clear synergistic effect was evident for other compounds. Most notably, dimethyl trisulfide reached an exceptionally high concentration only in the fermented-and-baked flour (Table 5), contributing a unique savory note that was not produced by either process alone. This indicates that fermentation establishes a distinct biochemical matrix which is then selectively and non-linearly modified by subsequent baking. The final aroma profile results from this sequenced interaction: fermentation provides the foundational aromatic compounds, while baking fine-tunes the profile through thermal reactions. The specific nutrient-rich matrix of buckwheat, which is richer in proteins and fats compared to leaf-based substrates, likely plays a key role in shaping this unique interactive outcome [4,67]. 

Although baking inherently leads to some loss of volatiles [2,68], the combined process of solid-state fermentation followed by baking effectively optimized the overall aroma by enhancing desirable notes and suppressing off-flavors. The resulting volatile profile was distinct from that achieved by either process applied in isolation, demonstrating how microbial fermentation and controlled thermal processing can be integrated to enhance the flavor complexity and acceptability of functional grain-based products.

#### 3.8.3. Impact of Baking and Fermentation on Aroma-Active Compounds of Cookies

To evaluate the influence of incorporating fermented flour on cookie aroma, the volatile profiles of three cookie groups were analyzed. 50 compounds with both significant content changes (|log_2_FC| > 1) and an rOAV > 1 were identified as key aroma contributors (Table 6). The commercial cookie (CM) served as a market reference, while the non-inoculated (NBC) and fermented buckwheat cookies (FBC) were directly comparable due to their matched formulation. Owing to inherent differences in recipe composition, comparisons with CM are descriptive of overall aroma profile differences rather than a direct quantitative benchmark.

Compared to CM, the addition of 20% buckwheat flour (whether fermented or not) introduced a shared set of key aroma-active compounds, establishing a distinct base profile. This was particularly evident among compounds with the highest rOAV values (>50) as in Table 6. The rOAV value of the following compounds increased remarkable in both NBS and FBS, such as the lipid-derived aldehydes (E,E)-2,4-decadienal and (2E,4Z)-2,4-decadienal, the easters butanoic acid, 3-methyl-, phenylethyl ester, and 5-hexyldihydro-2(3H)-furanone, the heterocyclic compound dihydro-5-pentyl-2(3H)-furanone, and the terpenoid α-irone. These substances are likely the key contributors to the aroma signature imparted by buckwheat flour and the butter added, regardless of fermentation. However, a direct comparison between FBC and NBC revealed the specific refinements brought by the combined process of *E. cristatum* fermentation and subsequent baking. While the core high-impact aroma profiles were similar, 30 compounds showed marked differences (|rOAV fold-change| > 2). Fermentation and baking uniquely up-regulated a suite of pleasant compounds in FBC, enriching the profile with sweet, floral, and fruity nuances contributed by compounds such as anethole, trans-anethole, citral, geraniol, 1-decanol, and decanal. Concurrently, distinct off-notes present in NBC—notably the burnt 2-ethyl-3,5-dimethylpyrazine and the animalic p-cresol—were absent or significantly down-regulated in FBC. Thus, the primary effect of fermentation was not to overhaul the core aroma set but to refine it by selectively enhancing desirable nuances and suppressing specific undesirable ones.

The volatile profile of the final cookies demonstrates that solid-state fermentation with *E. cristatum*, followed by baking, effectively refines the sensory quality of buckwheat-based products. This refinement is achieved through a dual mechanism: the up-regulation of key pleasant aroma compounds and the down-regulation of specific off-flavors. The significant enhancement of fruity, floral, and sweet notes in FBC—driven by compounds like butanoic acid, 3-methyl-, phenylethyl ester, anethole, and geraniol—alongside the suppression of musty and burnt off-notes (e.g., 2-ethyl-3,5-dimethylpyrazine, p-cresol), aligns with the documented flavor-modulating role of this fungus in other substrates, such as tea and fermented grains [4,54].

A comparison with the volatile changes in the fermented flour itself reveals both consistency and modulation by the food matrix [8,37,69]. The up-regulation of pleasant esters and aromatics (e.g., anethole) and the down-regulation of pyrazines are consistent trends observed in both the fermented flour (FBF) and the corresponding cookies (FBC), confirming the transfer and persistence of these microbial modifications through the baking process [39,70,71,72]. However, a key divergence is noted for some fermentation-specific markers. For instance, compounds strongly associated with fungal metabolism in flour, such as 1-octen-3-one and dimethyl trisulfur compounds, showed a less pronounced sensory impact (rOAV) in the final cookie [73,74,75,76,77]. This attenuation likely results from the moderating role of the complex food matrix and the baking process, where interactions with other ingredients (e.g., fats in butter, milk and gluten flour), participation in Maillard reactions, caramelization or partial volatilization during baking [78,79] can transform or mask certain labile compounds generated during fermentation.

In conclusion, the integration of *E. cristatum* fermentation into the production of buckwheat cookies creates a beneficial synergy with subsequent baking. While baking establishes the foundational roasted and fatty notes from the matrix, fermentation acts as a targeted biocatalytic pre-treatment that enriches the profile with desirable complexities and purges it of key defects. The final product aroma is therefore a result of this sequenced interaction, yielding a flavor profile that is more balanced and appealing than that achieved by either process alone.

#### 3.8.4. Antioxidant Activity of Cookies

Antioxidant activity is one of the key bioactivities of buckwheat [3,4]. To determine the effect of adding *E. cristatum*-fermented buckwheat flour on the antioxidant capacity of cookies, the DPPH, ABTS, OH radical scavenging activity, and Fe reduction ability were measured for three different types of cookies.

**Figure 6:**
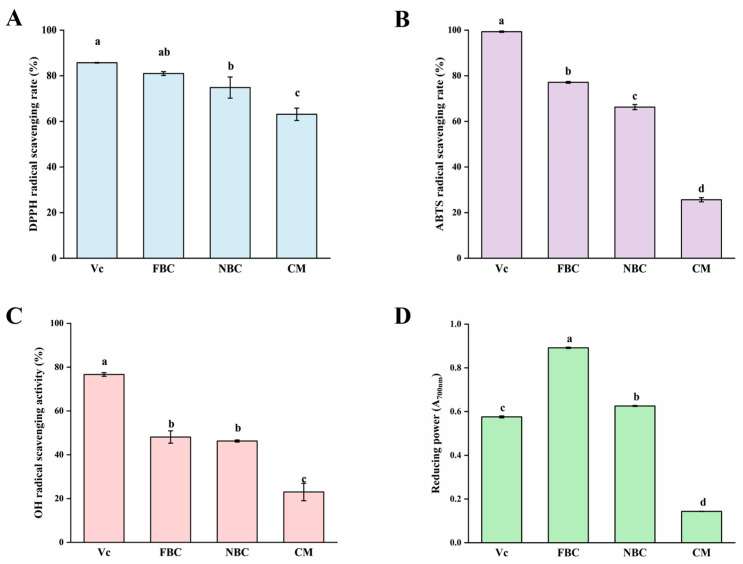
Comparison of antioxidant activity of different biscuits. (**A**) DPPH radical scavenging rate, (**B**) ABTS radical scavenging rate, (**C**) hydroxyl radical scavenging rate, (**D**) Fe reduction ability. The concentrations of Vc are 20 µg/mL (**A**), 1 mg/mL (**B**), 2 mg/mL (**C**), and 1 mg/mL (**D**), the concentration of cookies is 100 mg/mL. Different lowercase letters indicate significant differences, *p* < 0.05.

As shown in Figure 6, the antioxidant activity of both experimental buckwheat cookies was significantly higher than that of the commercial product. More notably, cookies made with fermented buckwheat flour demonstrated superior antioxidant capacity compared to those with the non-inoculated flour. Specifically, the fermented cookies showed a 16.4% increase in ABTS radical scavenging rate (Figure 6B) and a 42.5% greater ferric reducing power (Figure 6D). The lower antioxidant activity of commercially available buckwheat cookies may be due to their lower buckwheat content, or the flavonoid/polyphenol levels. The fermentation process with *E. cristatum* followed by baking increased the flavonoid and polyphenol content in buckwheat (Table 4), which likely contributed to the enhanced antioxidant activity of the fermented buckwheat cookies. This enhancement is likely attributed to the microbial enzymatic activity of *E. cristatum*, which can hydrolyze bonds conjugating phenolics to cell wall structures, thereby increasing the extractability and bioavailability of these antioxidant compounds [38,39].

## 4. Conclusions

This study demonstrates that incorporating 20% *E. cristatum*-fermented Tartary buckwheat flour improved the quality of cookies through the synergistic effects of solid-state fermentation and baking. The fermented cookies (FBC) exhibited enhanced texture, along with significantly higher levels of total flavonoids (4.97 mg/g), polyphenols (2.31 mg/g), and increased antioxidant activity compared to control samples. Volatile compound analysis showed that the combined process beneficially transformed the aroma profile. It enhanced key pleasant aroma-active compounds associated with sweet, floral, and fruity notes, while simultaneously suppressing undesirable off-aromas, such as those from certain pyrazines and aldehydes. A distinct synergistic effect was highlighted by the exclusive detection of dimethyl trisulfide at a high relative odor activity value in the fermented-and-baked flour. The improved texture was supported by microstructural analysis, which revealed a more homogeneous matrix in the FBC.

Regarding safety, while a comprehensive toxicological assessment is needed for commercialization, the preliminary profile is supported by the strain’s origin from traditionally fermented tea, the absence of detected mycotoxins in the fermented grains [4,79], effective pasteurization during high-temperature baking, and compliance of the cookies with standard food safety parameters [78]. A full safety evaluation remains a focus for subsequent studies.

In summary, solid-state fermentation with *E. cristatum* (CCTCC M 20231875) followed by baking proved effective for developing quality-enhanced buckwheat cookies, based on the specific combination with Tartary buckwheat ‘Chengku No. 2’. Future research should systematically evaluate diverse strain–variety pairs to optimize and generalize the process, alongside further safety validation, parameter optimization, and functional benefit assessment through targeted in vitro and in vivo studies. The synergistic interplay between fungal metabolism and thermal processing presents a promising pathway for upgrading the quality of grain-based products.

## Figures and Tables

**Table 1:** Table of sensory evaluation criteria.

Project	Evaluation Criteria	Score
Morphology (10)	The shape should be intact and aesthetically pleasing, with clear patterns. There should be no significant or minor concave areas or cracks.	8~10
	The shape is somewhat unappealing, with clear patterns. There are some cracks and minor concave areas.	5~7
	The shape is unattractive, with unclear patterns, significant cracking, and large concave areas.	0~4
Flavor profile (35)	The cookies exhibit a pleasant aroma, with a suitable buckwheat flavor, notes of fungi and chocolate, and a sweet yet not overly cloying taste. The texture is appropriately granular, and there are no off-flavors.	30~35
	The cookies have a noticeable biscuit aroma, with a subdued buckwheat flavor. The fungal and chocolate notes are slightly faint, and the taste is somewhat sweet or mild. The texture is coarse, but there are no off-flavors.	20~29
	There is no biscuit aroma, and neither buckwheat nor chocolate flavors are present. The taste may be overly sweet and cloying, or lacking sweetness altogether, with an overall discordant aroma.	0~19
Crispness and texture (20)	The texture is crisp and airy, not sticky, and refreshing. The sweetness or saltiness is well-balanced, with a pleasant aftertaste.	15~20
	The texture is soft and tender, non-sticky, and not greasy. The sweetness or saltiness is moderate, with a relatively dry aftertaste.	5~14
	The texture is either rigid or excessively crumbly and rough.	0~4
Color (20)	The surface displays the characteristic buckwheat hue, with a uniform light yellow to light brown color, free from burnt areas, and exhibits good gloss.	15~20
	The color is relatively even, with an average level of gloss.	10~14
	There is charring, with color that is either too dark or too light, accompanied by poor gloss.	0~9
Organizational structure (15)	The texture is loose and delicate, with an internal structure consisting of a fine, porous network	12~15
	The texture is somewhat loose and delicate, with a fine, porous structure; however, the porosity is uneven.	5~11
	The texture is rough and hard, non-greasy, with large cracks and significant cavities.	0~4

**Table 2:** Influence of fermented Tartary buckwheat flour content on the color parameters of cookies.

Cookie Color Intensity	The Ratio of Fermented Buckwheat Flour in the Total Flour Mix				
	0%	10%	20%	30%	40%
L*	70.18 ± 0.213 ^a^	56.86 ± 0.085 ^b^	54.28 ± 0.153 ^c^	52.28 ± 0.384 ^d^	49.37 ± 0.074 ^e^
a*	5.9 ± 0.055 ^b^	6.84 ± 0.076 ^a^	5.38 ± 0.026 ^dc^	4.91 ± 0.093 ^d^	5.68 ± 0.145 ^c^
b*	22.32 ± 0.236 ^a^	21.19 ± 0.102 ^b^	19.04 ± 0.107 ^c^	17.9 ± 0.196 ^d^	15.21 ± 0.153 ^e^

Note: L* indicates lightness and darkness, a* indicates redness-greenness; b* indicates blueness-yellowness. Different lowercase letters in the same column indicate a significant statistical difference at *p* < 0.05.

**Table 3:** Textural properties of cookies with *E. cristatum*-fermented and baked buckwheat flour.

Cookie Color Intensity	The Ratio of Fermented Buckwheat Flour in the Total Flour Mix				
	0%	10%	20%	30%	40%
Hardness	469.62 ± 28.47 ^c^	356.95 ± 4.93 ^d^	568.11 ± 8.51 ^b^	655.67 ± 13.16 ^a^	523.15 ± 18.23 ^b^
Chewiness	6.628 ± 1.23 ^c^	12.1 ± 0.49 ^b^	11.57 ± 0.96 ^b^	17.62 ± 1.88 ^ab^	14.05 ± 1.22 ^a^

Note: Different lowercase letters in the same column indicate a significant statistical difference at *p* < 0.05.

**Table 4:** Effects of different supplemental levels of Tartary buckwheat powder on nutritional components of fermented cookies.

Buckwheat Cookie Type	Water (g/100 g)	Fat (g/100 g)	Starch (g/100 g)	Crude Protein (g/100 g)	Total Flavonoids (mg/g)	Total Polyphenols (mg/g)
NBC	1.37 ± 0.13 ^a^	35.55 ± 0.13 ^a^	40.00 ± 0.30 ^b^	5.91 ± 0.02 ^b^	4.00 ± 0.05 ^b^	1.67 ± 0.07 ^b^
FBC	1.16 ± 0.16 ^ab^	37.35 ± 0.06 ^a^	34.10 ± 0.30 ^c^	5.31 ± 0.01 ^c^	4.97 ± 0.13 ^a^	2.31 ± 0.06 ^a^
CM	0.77 ± 0.05 ^b^	24.49 ± 1.02 ^b^	46.60 ± 0.89 ^a^	7.92 ± 0.02 ^a^	1.86 ± 0.09 ^c^	0.74 ± 0.03 ^c^

Note: Different lowercase letters in the same column indicate a significant statistical difference at *p* < 0.05.

**Table 5:** Main changed volatile aroma components in four types of buckwheat flour.

Class I	Compounds	rOAV				Odor
		NBF-U	NBF-B	FBF-U	FBF-B	
Alcohol	1-Octen-3-ol	4.54	25.34	199.84	65.87	fatty, fruity, grassy, mushroom, perfumy, sweet
	1-Nonanol	22.23	43.86	0.43	5.86	fresh, clean, fatty, floral, rose, orange, dusty, wet, oily
Aldehyde	(E,E)-2,6-Nonadienal	62.51	63.91	183.77	173.53	fresh, citrus, green, cucumber, melon
	(E,Z)-2,6-Nonadienal	3125.49	3195.45	9188.31	8676.61	cucumber, green
	(Z)-6-Nonenal	1966.82	1724.81	5031.87	3773.82	green, cucumber, melon, cantaloupe, honeydew, waxy, vegetable, orris, violet, leafy
	cis-7-Decen-1-al	18.26	14.24	39.84	33.23	citrus, aldehydic, cucumber
	(2E,4Z)-2,4-Decadienal	106.83	180.74	131.32	16.90	fried, fatty, geranium, green, waxy
	(Z,Z)-3,6-Nonadienal	868.55	758.55	2479.61	647.42	fatty, soapy, cucumber
	10-Undecenal	1.51	0.34	515.14	95.95	waxy, aldehydic, rose, mandarin, citrus, soapy, fatty
	2-Nonenal	142.24	135.43	525.13	12.04	fatty, green, waxy, cucumber, melon
	(E)-2-Nonenal	3976.63	2863.38	3995.25	110.80	fatty, green, cucumber, aldehydic, citrus
	(E)-4-Decenal	1.19	1.32	3.05	0.89	fresh, aldehydic, citrus, orange, mandarin, tangerine, green, fatty
	Benzaldehyde	2.62	4.16	1.31	4.35	sweet, bitter, almond, cherry
	Benzeneacetaldehyde	107.50	174.13	1147.99	73.02	floral, honey, rose, cherry
	Hexanal	2.07	2.31	3.02	13.54	aldehyde, grassy, green, leafy, vinegar
Aromatics	Anethole	3.27	1.03	1184.29	177.73	sweet, exotic, flowery, stewed
	1-ethyl-3-methyl-Benzene	10.45	16.85	6.66	20.58	-
	Styrene	3.59	4.71	21.96	5.74	penetrating, balsamic, gasoline
	Trans-Anethole	0.86	0.27	311.65	46.77	sweet, anisic, licorice, mimosa
Ester	2-Propenoic acid, ethyl ester	880.25	499.75	1027.19	1010.92	harsh, plastic, acrylate, fruity
	2-methyl-Butanoic acid, 2-methylpropyl ester	3.37	3.85	9.36	7.84	sweet, fruity
	Isobutyl isovalerate	4.26	4.87	11.83	9.92	sweet, fruity, apple, raspberry, green, banana
	5-hexyldihydro-2(3H)-Furanone	2.71	3.03	3.97	12.84	fresh, oily, waxy, peach, coconut, buttery, sweet
	2,2,4-Trimethyl-1,3-pentanediol diisobutyrate	8.03	0.14	8.22	276.48	-
	Benzoic acid, methyl ester	12.65	13.29	133.45	21.91	phenol, wintergreen, almond, floral, conga
	3-methyl-Butanoic acid, 2-phenylethyl ester	1840.41	1799.07	42.22	237.44	floral, fruity, sweet, rose, peach, apricot
Heterocyclic compound	2-Isobutylthiazole	31.46	33.40	78.35	67.56	green, wasabi, privet, tomato, leafy, earthy, vegetable, metallic
	tetrahydro-6-methyl-2H-Pyran-2-one	1.15	1.45	3.97	4.39	creamy, fruity, coconut
	2,3-dimethyl-Pyrazine	0.38	0.43	1.14	0.03	nutty, nut skin, cocoa, peanut, buttery, coffee, walnut, caramel, roasted
	2-ethyl-3,5-dimethyl-Pyrazine	6261.12	5242.38	357.60	81.08	burnt, almond, roasted, nutty, coffee
	3-ethyl-2,5-dimethyl-Pyrazine	29.12	24.38	1.66	0.38	potato, cocoa, roasted, nutty
	2-Acetylthiazole	2.87	6.43	9.34	8.11	nutty, popcorn, roasted, peanut, hazelnut
Hydrocarbons	Tridecane	2.92	2.77	12.24	2.64	alkane
Ketone	(E,E)-3,5-Octadien-2-one	14.45	16.73	2.72	1.76	fruity, green, grassy
	2,2-dimethyl-3-Hexanone	10.17	11.78	30.72	27.16	-
	4-Undecanone	3.12	3.02	6.42	8.10	fruity
	1-Octen-3-one	2360.90	11,080.32	23,867.87	16,919.64	mushroom
Nitrogen compounds	Dodecanenitrile	485.47	515.28	301.35	109.77	citrus, orange, peel, metallic, spicy
Phenol	2-methoxy-Phenol	151.46	156.05	7.42	3.29	nutty
	p-Cresol	41.16	32.14	1.09	9.50	phenol, narcissus, animalic, mimosa
Sulfur compounds	(isothiocyanatomethyl)-Benzene	1.89	10.56	96.71	214.07	mild, watercress, dusty, medicinal, horseradish, oily
	Dimethyl trisulfur compounds	4097.03	5961.14	8774.47	22,114.80	sulfury, cooked onion, savory, meaty
Terpenoids	D-Limonene	0.31	0.36	2.95	1.50	citrus
	(+)-alpha-Pinene	0.37	2.57	3.11	0.79	harsh, terpene, aromatic, minty
	alpha-Irone	0.77	0.86	1.12	3.63	orris, floral, berry, violet, woody, powdery
	beta-Ocimene	0.39	0.55	3.77	0.23	apple, pear, fruity
	3,7-dimethyl-, (Z)-1,3,6-Octatriene	0.39	0.55	3.77	0.23	warm, floral, herbal, flowery, sweet
	1-methyl-4-(1-methylethylidene)-Cyclohexene	0.37	0.36	1.84	0.45	citrus, pine
	Linalool	4.08	6.08	26.82	7.73	floral, green

Note: NBF-B, Non-inoculated, Baked Buckwheat Flour; NBF-U, Untreated, Non-inoculated Buckwheat Flour; FBF-U, Untreated, Fermented Buckwheat Flour; FBF-B, Fermented, Baked Buckwheat Flour.

**Table 6:** Main changed volatile aroma components in three types of cookies.

Class I	Compounds	rOAV			Odor
		CM	NBC	FBC	
Alcohol	4-Phenyl-2-butanol	1.29	1.05	3.14	floral, peony, foliage, sweet, mimosa, heliotrope
	(6Z)-Nonen-1-ol	26.17	20.41	45.96	fresh, green, melon, waxy, honeydew, cantaloupe, cucumber, clean
	6-Undecanol	6.54	3.00	7.29	-
	1-Decanol	6.56	2.28	6.99	fatty, waxy, floral, orange, sweet, watery
	1-Nonanol	27.40	4.19	3.44	fresh, clean, fatty, floral, rose, orange, dusty, wet, oily
Aldehyde	(2E,4Z)-2,4-Decadienal	141.64	4540.31	6043.79	fried, fatty, geranium, green, waxy
	(E,E)-2,4-Decadienal	676.92	13,731.43	12,646.00	dusty, waxy, oily, soapy
	Tridecanal	2.68	35.18	37.40	fresh, clean, aldehydic, soapy, citrus, petal, waxy, grapefruit, peel
	Benzeneacetaldehyde	140.76	450.01	286.02	floral, honey, rose, cherry
	Decanal	398.40	212.30	442.47	sweet, aldehydic, waxy, orange peel, citrus, floral
	(E)-2-Decenal	3.86	2.05	6.31	waxy, fatty, earthy, green, cilantro, mushroom, aldehydic, fried, chicken, fatty, tallow
	Piperonal	5.78	2.74	5.86	heliotrope, flowery, sweet, powdery, coconut, vanilla
	(Z,Z)-3,6-Nonadienal	1300.74	511.53	486.11	fatty, soapy, cucumber
	(E)-2-Nonenal	928.38	289.38	2600.41	fatty, green, cucumber, aldehydic, citrus
	2-Nonenal	100.91	31.45	29.62	fatty, green, waxy, cucumber, melon
	Hexanal	16.40	5.11	4.81	aldehyde, grassy, green, leafy, vinegar
	(E)-6-Nonenal	443.59	85.15	400.93	cucumber, fresh, green, fatty, waxy, iris, fruity, aldehydic
Aromatics	2,6-dimethyl-Naphthalene	0.19	1.51	2.11	grassy
	trans-Anethole	0.28	0.37	1.55	sweet, anisic, licorice, mimosa
	Anethole	1.06	1.40	5.87	sweet, exotic, flowery, stewed
	Styrene	14.08	3.78	2.07	penetrating, balsamic, gasoline
Ester	5-hexyldihydro-2(3H)-Furanone	4.30	377.88	491.93	fresh, oily, waxy, peach, coconut, buttery, sweet
	3-methy-l, 2-Dodecanoic acid, methyl ester	1.14	13.23	13.55	waxy, soapy, creamy, coconut, mushroom
	Butanoic acid, 3-methyl-, phenylethyl ester	229.02	1361.85	2615.38	floral, fruity, sweet, rose, peach, apricot
	2,2,4-Trimethyl-1,3-pentanediol diisobutyrate	1.00	2.19	7.52	-
	5-butyldihydro-2(3H)-Furanone	15.06	8.54	20.35	sweet, coconut, waxy, creamy, tonka, dairy, fatty
	Decanoic acid, methyl ester	207.45	105.03	223.47	oily, wine, fruity, floral
	2-Propenoic acid, ethyl ester	814.21	177.34	835.00	harsh, plastic, acrylate, fruity
Heterocyclic compound	dihydro-5-pentyl-2(3H)-Furanone	41.41	750.56	925.80	coconut, woody
	2-ethyl-3,5-dimethyl-Pyrazine	1677.17	1104.90	39.88	burnt, almond, roasted, nutty, coffee
	2,3-diethyl-5-methyl-Pyrazine	231.89	108.33	291.16	musty, nut skin, earthy, roasted, hazelnut, toasted, potato, dusty, foliage, vegetable
	4-methyl-5-Thiazoleethanol	1.04	0.47	1.14	fatty, cooked, beefy, juice
	5H-5-Methyl-6,7-dihydrocyclopentapyrazine	3.08	1.38	3.29	earthy, baked, potato, peanut, roasted
Ketone	5-Methyl-(E)-2-hepten-4-one	3639.83	4331.77	2152.48	hazelnut, nutty
	4-Undecanone	2.46	1.37	3.99	fruity
	Isophorone	14.08	1.40	1.83	cool, woody, sweet, green, camphor, fruity, musty, cedarwood, tobacco, leathery
Nitrogen compounds	Dodecanenitrile	35.03	67.32	95.20	citrus, orange, peel, metallic, spicy
Phenol	2-methoxy-Phenol	0.71	5.54	1.04	nutty
	3-ethyl-Phenol	3.57	15.11	4.65	musty
	p-Cresol	90.69	94.44	8.68	phenol, narcissus, animalic, mimosa
Sulfur compounds	Dimethyl trisulfur compounds	2488.97	7878.45	2593.25	sulfury, cooked onion, savory, meaty
Terpenoids	alpha-Irone	1.22	488.30	506.72	orris, floral, berry, violet, woody, powdery
	alpha-Farnesene	0.35	4.70	5.07	citrus, herbal, lavender, bergamot, myrrh, neroli, green
	beta-Phellandrene	0.43	0.65	1.41	terpenic, herbal
	Geraniol	1.58	0.94	2.18	sweet, floral, fruity, rose, waxy, citrus
	(E)-3,7-dimethyl-2,6-Octadienal	2.92	1.25	3.05	citrus, lemon
	Citral	1.20	0.44	1.29	sharp, lemon, sweet
	trans-Rose oxide	17.23	0.22	1.03	floral

Note: CM, Commercial Buckwheat Cookies; FBC, Fermented Buckwheat Cookies; NBC, Non-inoculated Buckwheat Cookies.

## Data Availability

The data supporting this study are provided within the article. Additional information is available from the corresponding author upon request.
